# Signature proteins that are distinctive of alpha proteobacteria

**DOI:** 10.1186/1471-2164-6-94

**Published:** 2005-06-16

**Authors:** Pinay Kainth, Radhey S Gupta

**Affiliations:** 1 Department of Biochemistry and Biomedical Sciences, McMaster University Hamilton, L8N 3Z5, Canada

## Abstract

**Background:**

The alpha (α) proteobacteria, a very large and diverse group, are presently characterized solely on the basis of 16S rRNA trees, with no known molecular characteristic that is unique to this group. The genomes of three α-proteobacteria, *Rickettsia prowazekii *(RP), *Caulobacter crescentus *(CC) and *Bartonella quintana *(BQ), were analyzed in order to search for proteins that are unique to this group.

**Results:**

Blast analyses of protein sequences from the above genomes have led to the identification of 61 proteins which are distinctive characteristics of α-proteobacteria and are generally not found in any other bacteria. These α-proteobacterial signature proteins are generally of hypothetical functions and they can be classified as follows: (i) Six proteins (CC2102, CC3292, CC3319, CC1887, CC1725 and CC1365) which are uniquely present in most sequenced α-proteobacterial genomes; (ii) Ten proteins (CC1211, CC1886, CC2245, CC3470, CC0520, CC0365, CC0366, CC1977, CC3010 and CC0100) which are present in all α-proteobacteria except the *Rickettsiales*; (iii) Five proteins (CC2345, CC3115, CC3401, CC3467 and CC1021) not found in the intracellular bacteria belonging to the order *Rickettsiales *and the *Bartonellaceae *family; (iv) Four proteins (CC1652, CC2247, CC3295 and CC1035) that are absent from various *Rickettsiales *as well as *Rhodobacterales*; (v) Three proteins (RP104, RP105 and RP106) that are unique to the order *Rickettsiales *and four proteins (RP766, RP192, RP030 and RP187) which are specific for the *Rickettsiaceae *family; (vi) Six proteins (BQ00140, BQ00720, BQ03880, BQ12030, BQ07670 and BQ11900) which are specific to the order *Rhizobiales*; (vii) Four proteins (BQ01660, BQ02450, BQ03770 and BQ13470) which are specific for the order *Rhizobiales *excluding the family *Bradyrhizobiaceae*; (viii) Nine proteins (BQ12190, BQ11460, BQ11450, BQ11430, BQ11380, BQ11160, BQ11120, BQ11100 and BQ11030 which are distinctive of the *Bartonellaceae *family;(ix) Six proteins (CC0189, CC0569, CC0331, CC0349, CC2323 and CC2637) which show sporadic distribution in α-proteobacteria, (x) Four proteins (CC2585, CC0226, CC2790 and RP382) in which lateral gene transfers are indicated to have occurred between α-proteobacteria and a limited number of other bacteria.

**Conclusion:**

The identified proteins provide novel means for defining and identifying the α-proteobacteria and many of its subgroups in clear molecular terms and in understanding the evolution of this group of species. These signature proteins, together with the large number of α-proteobacteria specific indels that have recently been identified , provide evidence that all species from this diverse group share many unifying and distinctive characteristics. Functional studies on these proteins should prove very helpful in the identification of such characteristics.

## Background

The α-proteobacteria comprise a large and extremely diverse group of Gram-negative bacteria which form a part of the largest known phyla within prokaryotes, namely the proteobacteria [1]. The vast diversity of the α-subdivision is clearly evident through the lifestyle differences among its members making them important in agricultural, medical and industrial fields. Such examples include the animal and human intracellular pathogens (*Rickettsia*, *Bartonella*, and *Brucella*) [1-3], the plant pathogens and symbiotic soil bacteria (*Agrobacterium*, *Sinorhizobium*, *Mesorhizobium*, and *Bradyrhizobium*) [1,4-6], the *Drosophila *endosymbiont (*Wolbachia*) [1] and a number of other free-living bacteria occupying a wide variety of ecological niches [1]. Furthermore, this group exhibits a wide spectrum of characteristics in terms of morphology (spiral, rod, stalked), metabolism (phototrophs, heterotrophs, and chemolithotrophs), physiology and cell division mechanisms [1,7,8]. In addition to their great diversity in these regards, this group of species is also of central importance due to compelling evidence indicating that a large proportion of the genes in eukaryotic cells, especially those related to mitochondria, have an α-proteobacterial ancestry [9-16].

In the current view, the α-subdivision are thought to form a more recently branching monophyletic taxon emerging after the epsilon and delta but before the beta and gamma subdivisions or Classes of proteobacteria [1,13,17]. Although this group is distinguished from other major bacterial groups based on 16S rRNA and other gene phylogenies [7,13,17-19], no set of criteria exists to clearly define and circumscribe the α-proteobacteria in clear and unambiguous molecular terms [1]. Thus, the following question remains: what defining molecular characteristics distinguish an α-proteobacterium and its subgroups from all other bacteria? The task of identifying such markers is aided by the availability of 18 completely sequenced α-proteobacterial genomes along with 10 partially sequenced genomes [11,20-33], belonging to the following orders: *Rhizobiales*, *Rickettsiales*, *Caulobacterales*, *Rhodobacterales*, *Sphingomonadales *and *Rhodospirillales *[34]. The comparative analyses of genomes provides a valuable resource and a very powerful means for identifying characteristics that are unique to a particular group of species [6,16,27,28,32,35,36]. We have used these data to identify a large number of conserved inserts and deletions (indels) in protein sequences that are distinctive characteristics of different groups of bacteria and provide molecular means for their identification and characterization [13,37-40]. Recently, we have also identified many conserved indels in protein sequences that are useful for defining the α-proteobacteria group, and its various subgroups, in molecular terms [17]. The distribution pattern of these signatures in different α-proteobacteria has been used to deduce a working model to describe the interrelationships as well as the branching order among the α-proteobacteria species [17].

In the present study, a new type of taxonomic marker is described which provides an additional means to define the α-proteobacteria group as well as the relationship within this group. These new markers consist of whole proteins that are specific to certain groups or subgroups of bacteria and are not found in any other phyla [35]. In this work we have identified a large number of proteins which are specific to either the α-proteobacteria group as a whole or its various subgroups. These signature proteins were identified in BLASTP searches [41] of individual proteins from the genomes of three α-proteobacterial species (viz. *Rickettsia prowazekii, Caulobacter crescentus *and *Bartonella quintana*) [11,24,32], which show important differences in lifestyles and physiology. Results of this study presented here will prove useful in developing a clearer picture of α-proteobacterial phylogeny as well as aid in the identification of bacterial strains belonging to this group and its subgroups. Functional studies on these α-proteobacteria specific proteins should prove instrumental in the discovery of novel physiological characteristics that are uniquely shared by members of this large and diverse group of bacteria.

## Results

These studies were undertaken with the aim of identifying proteins that are uniquely found in α-proteobacteria and which could provide novel molecular means for defining and identifying bacteria belonging to this group and its subgroups. To identify proteins which are specific to α-proteobacteria or its subgroups, BLAST searches were carried out individually on every single annotated protein present in the genomes of three different α-proteobacteria, *C. crescentus*, *R. prowazekii *and *B. quintana*. These genomes were chosen because of their different sizes (*R. prowazekii*, 1.11 Mb with 835 open reading frames (ORFs); *B. qunitana*, 1.58 Mb, 1142 ORFs; *C. crescentus*, 4.02 Mb with 3737 ORFs) and because these species display important differences in life-style and other characteristics [11,24,32]. Results of the BLAST searches were inspected in order to identify proteins which are only found in α-proteobacteria, as well as proteins where the only acceptable BLAST scores as indicated by their expected values (E values) were from α-proteobacteria [41]. These studies have resulted in the identification of 61 signature proteins, which appear distinctive of α-proteobacteria and are generally not found in any other *Bacteria*. For all of these proteins, the lengths of the query proteins as well as the E values obtained from BLAST searches for different hits are shown (Tables 1, 2, 3, 4, 5, 6, 7, 8, 9). The former values are important in determining the significance of the observed BLAST scores (See Methods section). Additionally, for all of the α-proteobacteria specific proteins, the length of the hit protein over the query sequence is shown in brackets to show that the homologues in different species are of similar length. Most of the α-proteobacterial signature proteins that we have identified are of hypothetical function as annotated in the NCBI database . For the sake of presentation and discussion, we have arbitrarily divided these proteins into ten groups based on their distribution patterns among α-proteobacteria.

**Table 1:** Signature Proteins Specific for the Alpha-proteobacteria.^a^

**Protein**	**CC2102 **[16126341]	**CC3292 **[16127522]	**CC3319^b ^**[16127549]	**CC1887^c ^**[16126130]	**CC1725 **[16125969]	**CC1365 **[16125614]
**Length**	**162**	**224**	**89**	**105**	**100**	**161**
*Mag. mag*.	2e-19 (1.04)	9e-49 (1.29)	2e-15 (0.99)	3e-20 (1.10)	1e-08 (0.88)	2e-21 (0.87)
*Rhod. rubr*.	3e-17 (1.09)	1e-42 (0.92)	1e-12 (1.04)	4e-21 (1.12)	1e-08 (1.09)	3e-17 (0.96)
*Nov. aro*.	1e-19 (1.02)	3e-45 (1.04)	4e-07 (0.88)	2e-25 (1.30)	-	1e-11 (0.98)
Z. mobilis *	4e-24 (1.01)	6e-40 (1.00)	-	-	-	5e-11 (1.02)
C. cres.*	1e-64 (1.00)	e-106 (1.00)	1e-34 (1.00)	3e-61 (1.00)	4e-49 (1.00)	7e-79 (1.00)
Sil. pom.*	3e-16 (0.93)	2e-44 (1.15)	8e-11 (0.97)	3e-22 (1.21)	8e-06 (0.99)	4e-12 (0.96)
*Sil. sp*.	1e-13 (0.98)	1e-43 (1.14)	8e-09 (0.99)	5e-24 (1.22)	1e-05 (0.88)	9e-12 (0.89)
*Rh. spha*.	1e-16 (0.94)	3e-43 (1.11)	4e-08 (0.94)	8e-24 (1.18)	2e-08 (1.00)	1e-11 (0.91)
Bra. jap.*	5e-21 (1.06)	8e-47 (1.11)	2e-12 (1.01)	2e-29 (1.29)	2e-09 (1.05)	2e-11 (1.15)
Rho. pal.*	8e-22 (1.10)	4e-45 (1.06)	7e-13 (1.45)	2e-25 (1.30)	2e-08 (1.05)	2e-12 (1.00)
Agr. tum.*	4e-21 (1.04)	7e-49 (1.02)	4e-10 (0.96)	3e-26 (1.26)	5e-06 (1.14)	7e-13 (1.10)
Sino. meli. *	4e-23 (1.06)	2e-49 (1.04)	6e-12 (1.01)	2e-22 (1.25)	5e-05 (0.93)	1e-14 (1.04)
Bru. mel.*	5e-19 (1.17)	4e-48 (1.02)	1e-12 (1.03)	1e-23 (1.30)	1e-07 (1.09)	4e-15 (1.04)
Bru. suis*	5e-19 (1.17)	4e-48 (1.02)	2e-12 (0.97)	1e-23 (1.30)	2e-08 (1.09)	4e-15 (1.07)
Meso. loti*	5e-18 (1.10)	9e-49 (1.00)	9e-18 (0.97)	9e-23 (1.26)	4e-09 (1.20)	3e-14 (1.40)
*Meso. sp*.	2e-17 (1.09)	2e-47 (1.03)	5e-16 (0.98)	6e-27 (1.26)	8e-06 (1.05)	7e-15 (1.33)
B. henselae*	8e-14 (1.12)	5e-41 (1.04)	4e-12 (0.96)	2e-23 (1.26)	5e-08 (1.09)	4e-10 (0.99)
B. Quintana*	5e-14 (1.12)	8e-42 (1.02)	4e-12 (0.96)	5e-21 (1.26)	1e-08 (1.09)	3e-11 (0.99)
R. conorii*	5e-10 (0.97)	3e-41 (0.83)	1e-07 (0.88)	1e-05 (0.94)	5e-11 (1.01)	7e-07 (0.98)
R. prowazekii*	4e-08 (0.98)	4e-40 (0.83)	3e-07 (0.88)	5e-07 (0.95)	2e-12 (1.07)	8e-06 (0.95)
R. typhi*	1e-08 (0.98)	2e-40 (0.83)	3e-07 (0.88)	4e-07 (0.95)	5e-11 (1.07)	3e-06 (0.98)
*R. akari*	6e-09 (0.97)	9e-41 (0.90)	1e-07 (0.88)	2e-05 (0.94)	7e-12 (1.07)	3e-07 (0.98)
*R. rickettsii*	1e-10 (0.97)	9e-41 (0.83)	1e-07 (0.88)	1e-05 (0.94)	5e-11 (1.01)	7e-07 (0.98)
*R. sibirica*	2e-10 (0.97)	5e-41 (0.83)	1e-07 (0.88)	1e-05 (0.94)	5e-11 (1.01)	3e-07 (0.98)
Wolbachia*	5e-11 (0.90)	3e-20 (0.89)	2e-07 (0.99)	1e-08 (0.99)	1e-05 (1.04)	3e-04 (0.70)
Ana. mar.*	2e-09 (0.98)	2e-40 (0.92)	-	-	9e-07 (1.19)	-
*Ehr. canis*	2e-07 (1.01)	6e-39 (0.95)	2e-06 (1.04)	5e-10 (0.94)	-	0.023 (0.90)
Ehr. rum.*	1e-08 (0.94)	2e-39 (0.99)	2e-05 (1.07)	8e-11 (0.89)	-	4e-05 (0.84)
Non-Alpha	-	*Strep. glau*. 1.6 (1.89)	*Fuso. nucl*. 2.9 (3.87)	*Burk. fung*. (3.69)	*Azo. sp*. 0.44 (0.91)	*Myc. pneum*. 0.98 (2.63)

^a ^Alpha-specific proteins were identified by BLAST searches on individual protein sequences on three α-proteobacterial genomes as described in the Methods section. The expected (E) values for various alpha-proteobacteria species as well as the first non-alpha species in the BLAST results are shown here. The values in brackets after the E values represent the ratios of the length of the hit protein divided by the query protein and a value close to 1.0 indicates that the homologues are of similar lengths. The CC numbers indicate the protein identification number in the *C. crescentus *genome. GenBank accession numbers for the query sequence are shown in square brackets. An asterisk (*) identifies bacterial genomes which are completely sequenced, whereas other sequences are from partially or incompletely sequenced genomes. Proteins not found in a given species are indicated with a dash (-). Abbreviations: *Agr. tum., Agrobacterium tumefaciens; Ana. mar., Anaplasma marginale; Azo. sp., Azoarcus sp. EbN1; B. henselae, Bartonella henselae; B. quintana, B. quintana; Bra. jap., Bradyrhizobium japonicum; Bru. mel., Brucella melitensis; Burk. fung., Burkholderia fungorum; C. cres., Caulobacter crescentus; Ehr. canis, Ehrlichia canis; Ehr. rum., Ehr ruminantium; Fuso. nucl., Fusobacterium nucleatum; Mag. mag., Magnetospirillum magnetotacticum; Meso., Mesorhizobium; Myc. pneum., Mycoplasma pneumoniae; Nov. aro., Novosphingobium aromaticivorans; Rhod. rubr., Rhodospirillum rubrum; Rh. spha., Rhodobacter sphaeroides; Rho. pal., Rhodopseudomonas palustris; Sil. pom., Silicibacter pomeroyi; Sil. sp., Silicibacter sp. TM1040; Sino. meli., Sinorhizobium meliloti; Strep. glau., Streptomyces glaucescens; Wolbachia, Wolbachia endosymbiont of Drosophila melanogaster*.

^b ^*Magnetococcus sp. MC-1 *(unclassfied) found in BLAST search with E value of 3e-09 [48832993].

^c ^Protein also found in *Eukaryotes*. The E values for a few representative eukaryotic species are as follows: *Homo sapiens*; 8e-10 [30583279], *Chlamydomonas reinhardtii*; 3e-09 [34334022], *Caenorhabditis elegans*; 5e-06 [7332202].

**Table 2:** Signature Proteins specific for Alpha-proteobacteria, except *Rickettsiales*.^a^

**Protein**	**CC1211 **[16125461]	**CC1886 **[16126129]	**CC2245 **[16126484]	**CC3470 **[16127700]	**CC0520^b ^**[16124775]
**Length**	**167**	**223**	**190**	**253**	**284**

*Mag. mag*.	3e-19 (1.09)	-	3e-16 (0.96)	5e-23 (0.97)	2e-38 (0.89)
*Rhod. rubr*.	3e-17 (0.73)	1e-07 (0.73)	3e-22 (0.64)	5e-04 (0.82)	2e-36 (0.93)
*Nov. aro*.	9e-13 (1.41)	3e-05 (0.91)	7e-13 (1.24)	6e-20 (0.84)	-
*Z. mobilis*	8e-12 (1.38)	-	5e-12 (1.21)	1e-15 (0.82)	-
*C. cres*.	5e-93 (1.00)	3e-58 (1.00)	1e-75 (1.00)	e-115 (1.00)	e-146 (1.00)
*Sil. pom*.	9e-21 (1.19)	2e-04 (0.53	7e-16 (1.04)	2e-10 (0.78)	5e-27 (0.88)
*Sil. sp*.	8e-20 (1.17)	5e-07 (0.65)	1e-15 (1.03)	3e-07 (0.78)	1e-27 (0.87)
*Rh. spha*.	1e-19 (1.24)	2e-06 (0.55)	2e-16 (1.09)	8e-07 (0.78)	2e-30 (0.92)
*Bra. jap*.	4e-20 (1.01)	1e-10 (1.61)	1e-25 (0.89)	5e-17 (0.94)	2e-44 (0.87)
*Rho. pal*.	1e-18 (1.28)	5e-10 (1.48)	3e-25 (1.12)	2e-17 (0.86)	5e-45 (0.91)
*Agr. tum*.	-	2e-10 (0.65)	-	1e-15 (0.85)	8e-46 (1.01)
*Sino. meli*.	1e-19 (1.04)	3e-12 (0.67)	4e-25 (0.91)	7e-16 (0.87)	1e-45 (0.89)
*Bru. mel*.	4e-22 (1.11)	2e-10 (0.68)	1e-26 (0.97)	1e-18 (0.82)	2e-41 (1.08)
*Bru. suis*	4e-22 (1.08)	2e-10 (0.70)	5e-26 (0.95)	8e-18 (0.83)	1e-41 (0.91)
*Meso. loti*	4e-24 (1.02)	5e-09 (0.89)	1e-27 (0.90)	8e-21 (0.83)	1e-44 (0.92)
*Meso. sp*.	1e-21 (1.07)	2e-10 (0.55)	5e-25 (0.94)	1e-18 (0.83)	1e-42 (0.91)
*B. henselae*	6e-20 (1.06)	9e-14 (0.63)	9e-22 (0.93)	5e-15 (0.84)	1e-28 (0.86)
*B. quintana*	2e-19 (1.06)	3e-12 (0.63)	3e-22 (0.93)	2e-11 (0.84)	2e-27 (0.89)
Non Alpha	*Kin. radio*. 0.097 (3.89)	*Vib. para*. 7.7 (0.82)	*Pse. aeruginosa *4.2 (1.88)	*Vib. cholerae *0.035 (1.96)	*Polaromonas sp*. 0.003 (0.95)
					
**Protein**	**CC0365 **[16124620]	**CC0366^c ^**[16124621]	**CC1977^d ^**[16126220]	**CC3010^e ^**[16127240]	**CC0100 **[16124355]

**Length**	**169**	**177**	**241**	**216**	**576**

*Mag. mag*.	2e-06 (1.05)	2e-06 (0.93)	1e-32 (0.98)	4e-24 (0.90)	7e-34 (0.92)
*Rhod. rubr*.	2e-08 (1.08)	4e-06 (0.91)	2e-36 (1.07)	8e-18 (0.94)	-
*Nov. aro*.	3e-07 (1.09)	3e-05 (0.93)	5e-29 (0.95)	3e-13 (0.91)	5e-26 (1.09)
*Z. mobilis*	-	-	1e-35 (0.97)	6e-12 (0.96)	2e-35 (1.02)
*C. cres*.	1e-44 (1.00)	1e-44 (1.00)	e-111 (1.00)	6e-93 (1.00)	0.0 (1.00)
*Sil. pom*.	4e-09 (1.12)	1e-07 (1.02)	2e-40 (0.99)	1e-19 (0.95)	2e-34 (0.99)
*Sil. sp*.	4e-08 (1.09)	1e-11 (1.02)	6e-38 (0.97)	2e-18 (0.96)	1e-33 (1.00)
*Rh. spha*.	1e-06 (1.09)	1e-08 (0.90)	6e-36 (0.98)	2e-20 (0.98)	7e-30 (0.98)
*Bra. jap*.	8e-07 (0.95)	2e-12 (1.06)	1e-28 (1.10)	8e-20 (0.94)	3e-35 (1.02)
*Rho. pal*.	9e-06 (0.96)	9e-10 (1.05)	2e-33 (1.08)	2e-19 (0.99)	5e-35 (1.09)
*Agr. tum*.	1e-06 (0.95)	7e-10 (1.21)	2e-35 (1.10)	3e-20 (0.98)	8e-35 (0.98)
*Sino. meli*.	2e-08 (0.95)	4e-10 (1.15)	3e-38 (1.08)	2e-20 (0.84)	2e-31 (0.94)
*Bru. mel*.	0.002 (0.84)	1e-10 (1.02)	1e-32 (1.08)	4e-20 (0.97)	4e-36 (0.99)
*Bru. suis*	2e-07 (0.94)	1e-10 (1.18)	8e-33 (1.08)	4e-20 (0.97)	5e-36 (0.94)
*Meso. loti*	1e-08 (0.96)	8e-08 (1.09)	-	5e-22 (0.95)	2e-34 (0.92)
*Meso. sp*.	7e-11 (0.94)	5e-11 (1.09)	1e-27 (1.09)	6e-23 (0.95)	1e-34 (0.99)
*B. henselae*	1e-06 (0.97)	3e-08 (1.06)	1e-27 (1.08)	2e-20 (0.95)	2e-27 (0.99)
*B. quintana*	5e-07 (0.97)	1e-07 (1.06)	2e-29 (1.08)	2e-20 (0.94)	4e-30 (0.99)
Non-Alpha	*Myc. galli*. 1.1 (1.17)	*Rhodo. baltica *0.005 (1.47)	*M. thermo*. 0.28 (1.03)	*Syn. elongatus *0.002 (1.33)	*Coryn. efficiens *0.11 (0.52)

^a ^Abbreviations and other details regarding BLAST results can be found in Table 1. E values of 0.0 indicate an extremely high degree of similarity between protein sequences. Additional abbreviations: *Coryn., Corynebacterium; Kin. radio., Kineococcus radiotolerans; M. thermo., Moorella thermoacetica; Myc. galli., Mycoplasma gallisepticum; Pse., Pseudomonas; Rhodo., Rhodopirellula; Syn., Synechococcus; Vib. para., Vibrio parahaemolyticus*.

^b,c ^*Magnetococcus sp. MC-1 *(unclassified) contains homologues of both proteins with E values of 1e-18 [48832519] and 8e-06 [48833234] respectively.

^d ^Protein also found in *Eukaryotes *with examples from representative species as follows: *Homo sapiens*; 1e-22 [21735485], *Oryza sativa*; 2e-18 [50939575] and *Cryptococcus neoformans*; 3e-17 [57225838].

^e ^One BLAST hit is *Pseudomonas sp*. with E value of 8e-20 [94976].

**Table 3:** Alpha-proteobacteria specific proteins which are absent in the *Rickettsiales *as well as (A) the *Bartonellaceae *family, or (B)the *Rhodobacterales *^a^

**Protein**	**CC2345 **[16126584]	**CC3115 **[16127345]	**CC3401 **[16127631]	**CC3467 **[16127697]	**CC1021 **[16125273]
**Length**	**159**	**136**	**120**	**152**	**130**

*Mag. mag*.	2e-38 (0.99)	2e-29 (0.90)	3e-14 (1.03)	7e-16 0.86	3e-14 (1.16)
*Rhod. rubr*.	1e-39 (1.01)	-	-	-	-
*Nov. aro*.	4e-35 (1.03)	8e-32 (1.08)	1e-09 (1.23)	1e-19 (1.10)	5e-06 (1.13)
*C. cres*.	2e-86 (1.00)	9e-79 (1.00)	8e-56 (1.00)	3e-82 (1.00)	1e-57 (1.00)
*Sil. pom*.	2e-38 (1.01)	1e-09 (0.96)	3e-09 (1.08)	5e-23 (0.99)	3e-14 (1.00)
*Sil. sp*.	3e-40 (1.00)	5e-10 (1.05)	5e-10 (1.19)	5e-24 (1.34)	8e-16 (1.10)
*Rh. spha*.	6e-40 (0.99)	1e-07 (1.00)	9e-07 (1.13)	1e-25 (1.02)	4e-10 (1.05)
*Bra. jap*.	2e-44 (1.04)	4e-27 (0.98)	6e-11 (1.22)	5e-28 (1.07)	6e-17 (1.12)
*Rho. pal*.	2e-45 (1.03)	8e-24 (0.96)	5e-13 (1.09)	5e-27 (1.05)	9e-15 (1.09)
*Agr. tum*.	7e-44 (0.99)	6e-30 (0.94)	2e-15 (1.07)	3e-28 (1.06)	1e-10 (0.98)
*Sino. meli*.	3e-44 (0.99)	1e-25 (0.83)	4e-14 (1.06)	7e-30 (1.14)	7e-13 (1.16)
*Bru. mel*.	2e-43 (1.01)	1e-28 (1.12)	3e-15 (1.27)	9e-29 (1.08)	1e-10 (1.17)
*Bru. suis*	2e-43 (1.01)	4e-29 (0.93)	2e-15 (1.06)	9e-29 (1.08)	1e-11 (1.18)
*Meso. loti*	1e-43 (1.01)	1e-28 (0.98)	3e-14 (1.11)	2e-27 (1.12)	5e-14 (1.15)
*Meso. sp*.	3e-43 (1.01)	1e-22 (0.79)	4e-10 (1.20)	5e-30 (1.09)	5e-13 (1.11)
Non-Alpha	*Vib. para*. 2.1 (1.04)	*Symbio. therm*. 1.3 (0.89)	*Burk. cepacia *0.96 (2.53)	*Noc. farcinica *0.17 (1.76)	*Pse. syringae *1.7 2.35
					
**Protein**	**CC1652 **[16125898]	**CC2247 **[16126486]	**CC3295 **[16127525]	**CC1035 **[16125287]	

**Length**	**250**	**46**	**169**	**224**	

*Mag. mag*.	2e-07 (0.91)	2e-06 (1.61)	2e-09 (0.52)	-	
*Rhod. rubr*.	7e-08 (1.01)	-	-	-	
*Nov. aro*.	-	7e-05 (1.41)	5e-06 (0.99)	3e-33 (1.19)	
*Z. mobilis*	-	1e-04 (1.65)	-	-	
*C. cres*.	7e-99 (1.00)	5e-21 (1.00)	3e-92 (1.00)	e-103 (1.00)	
*Bra. jap*.	5e-12 (0.87)	7e-07 (1.63)	6e-29 (1.00)	7e-43 (0.90)	
*Rho. pal*.	5e-12 (0.92)	5e-06 (2.41)	2e-27 (1.00)	3e-42 (0.97)	
*Agr. tum*.	4e-05 (1.00)	1e-04 (1.67)	3e-26 (1.01)	1e-39 (0.88)	
*Sino. meli*.	2e-09 (0.89)	7e-04 (1.67)	3e-24 (1.16)	2e-35 (0.89)	
*Bru. mel*.	2e-11 (0.89)	0.008 (1.70)	3e-28 (1.04)	4e-39 (0.88)	
*Bru. suis*	1e-11 (0.89)	0.008 (1.70)	2e-28 (0.99)	4e-39 (0.88)	
*Meso. loti*	2e-06 (0.88)	5e-05 (1.67)	9e-24 (1.05)	1e-38 (0.93)	
*Meso. sp*.	4e-11 (0.89)	2e-04 (1.63)	2e-25 (1.04)	2e-38 (0.80)	
*B. henselae*	1e-06 (0.89)	0.030 (1.70)	3e-18 (1.02)	1e-34 (0.88)	
*B. quintana*	2e-06 (0.89)	0.002 (1.70)	8e-18 (1.02)	2e-35 (0.88)	
Non-Alpha	*Meth. flagellatus *0.17 (1.53)	-	*Burk. cepacia *0.13 (2.89)	*Bdell. bacter*. 0.70 3.14	

^a ^The manner in which these alpha specific proteins were identified is as described in Table 1. Additional abbreviations: *Bdell. bacter., Bdellovibrio bacteriovorus; Burk., Burkholderia; Meth., Methylobacillus; Noc., Nocardia; Pse., Pseudomonas; Symbio. therm., Symbiobacterium thermophilum; Vib. para., Vibrio parahaemolyticus*.

**Table 4:** Signature Proteins Specific for the *Rickettsiales *or the *Rickettsiaceae *family^a^

**Protein**	**RP104^b ^**[15603981]	**RP105 **[15603982]	**RP106^c ^**[15603983]	**RP766 **[15604600]	**RP192 **[15604066]	**RP030 **[15603909]	**RP187^d ^**[15604061]
**length**	**1124**	**672**	**971**	**92**	**128**	**219**	**194**

*R. prowazekii*	0.0 (1.00)	0.0 (1.00)	0.0 (1.00)	8e-37 (1.00)	2e-41 (1.00)	e-118 (1.00)	e-108 (1.00)
*R. conorii*	0.0 (0.88)	0.0 (0.98)	0.0 (0.99)	3e-33 (1.18)	3e-30 (0.93)	e-107 (1.01)	e-100 (2.56)
*R. typhi*	0.0 (1.01)	0.0 (1.00)	0.0 (1.00)	1e-27 (0.85)	3e-40 (1.00)	e-116 (1.00)	e-102 (2.56)
*R. akari*	0.0 (0.90)	0.0 (1.00)	0.0 (1.01)	2e-25 (0.85)	2e-35 (1.02)	e-108 (1.01)	3e-99 (2.56)
*R. rickettsii*	0.0 (0.88)	0.0 (0.98)	0.0 (0.99)	4e-27 (0.85)	6e-32 (0.93)	e-109 (1.00)	e-99 (2.56)
*R. sibirica*	0.0 (0.88)	0.0 (0.98)	0.0 (0.99)	4e-27 (0.85)	2e-31 (0.93)	e-106 (1.01)	e-101 (2.56)
*Ehr. canis*	7e-22 (0.75)	4e-36 (1.25)	2e-26 (1.49)	-	-	-	-
*Ehr. rum*.	2e-22 (0.73)	2e-37 (1.22)	3e-23 (1.57)	-	-	-	-
*Ehr. chaf*.	5e-23 (0.73)	8e-36 (1.23)	-	-	-	-	-
*Wolbachia*	1e-22 (0.78)	7e-27 (1.27)	3e-20 (0.82)	-	-	-	-
*Ana. mar*.	2e-17 (0.78)	4e-25 (1.31)	2e-24 (1.05)	-	-	-	-
Non-*Rickettsiales*	*Meso. loti *0.004 (0.32)	*Sil. sp*. 0.002 (0.53)	*Xyl. fast*. 2e-07 (0.36)	*Leg. pneu*. 1.3 (2.60)	*M. thermo*. 8.1 (2.47)	*Myc. pulm*. 0.001 (4.72)	*Camp. lari *0.69 (4.09)

^a ^*Rickettsiale *and *Rickettsia *specific proteins were identified by whole-genome BLAST searches using protein sequences as probes from the fully sequenced *R. prowazekii *genome. The RP numbers refer to the protein identification number in the *R. prowazekii *genome. Other details are as in Table 1 and in Methods section. Additional abbreviations: *Camp., Campylobacter; Ehr. chaf., Ehrlichia chaffeensis; Leg. pneu., Legionella pneumophila; M. thermo., Moorella thermoacetica; Myc. pulm., Mycoplasma pulmonis; Xyl. fast., Xylella fastidiosa*.

^b,c^BLAST hits for the family *Anaplasmataceae *do not show homology over the entire range of the protein and may represent a conserved protein domain.

^d^BLAST hits for other *Rickettsia *strains are longer (497 aa) but contain a region that is almost completely homologous to the query sequence.

**Table 5:** Signature Proteins Specific for the *Rizobiales *order^a^

**Protein**	**BQ00140 **[49473701]	**BQ00720 [49473755]**	**BQ03880 **[49474026]	**BQ12030 **[49474691]	**BQ07670 **[49474353]	**BQ11900 **[49474679]
**Length**	**222**	**83**	**198**	**91**	**336**	**172**

*Bra. jap*.	5e-18 (1.10)	2e-09 (1.08)	2e-20 (0.97)	2e-07 (1.05)	5e-46 (1.06)	1e-17 (0.98)
*Rho. pal*.	3e-14 (1.11)	3e-09 (1.06)	2e-18 (0.97)	1e-07 (1.37)	-	-
*Agr. tum*.	5e-13 (1.06)	6e-11 (1.02)	1e-26 (0.98)	2e-12 (1.03)	7e-66 (0.97)	2e-24 (1.37)
*Sino. meli*.	3e-20 (1.00)	2e-13 (1.02)	7e-23 (0.98)	2e-14 (0.98)	5e-62 (1.02)	-
*Bru. mel*.	9e-39 (0.98)	4e-19 (1.04)	1e-38 (0.97)	3e-13 (0.54)	8e-70 (0.96)	2e-26 (1.02)
*Bru. suis*	4e-39 (0.98)	4e-19 (0.96)	1e-38 (1.03)	6e-20 (0.99)	8e-70 (0.98)	6e-27 (0.98)
*Meso. loti*	3e-25 (1.07)	2e-13 (1.18)	1e-25 (0.98)	4e-16 (0.99)	2e-67 (0.95)	1e-26 (1.03)
*Meso. sp*.	7e-18 (1.06)	2e-13 (1.02)	2e-25 (0.98)	2e-14 (1.02)	3e-61 (0.93)	7e-31 (1.02)
*B. henselae*	1e-92 (0.97)	6e-43 (1.00)	5e-92 (1.00)	2e-39 (1.00)	e-158 (1.01)	3e-81 (1.00)
*B. Quintana*	e-127 (1.00)	4e-43 (1.00)	e-107 (1.00)	1e-43 (1.00)	0.0 (1.00)	1e-91 (1.00)
Non – Rizobiale	*Bdell. bacter*. 0.25 (1.77)	*Sil. sp*. 0.46 (0.82)	*Vibrio fischeri *0.005 (2.49)	*St. pyogenes *0.12 (0.86)	*St. agalactiae *0.38 (2.65)	*Croc. watsonii *6.1 (2.85)

^a^Signature proteins that are distinctive of the order *Rhizobiales *were identified by carrying out BLAST searches of all proteins found in the genome of *B. quintana*. The BQ numbers refer to the protein identification number in the *B. quintana *genome. Abbreviations and further details regarding BLAST results are as in Table 1 and the Methods section.. Additional abbreviations: *Bdell. bacter., Bdellovibrio bacteriovorus; Croc., Crocosphaera; St., Streptococcus*.

**Table 6:** Signature Proteins specific for the *Rizobiales *except the *Bradyrhizobiaceae *family^a^

**Protein**	**BQ01660 **[49473833]	**BQ02450 **[49473907]	**BQ03770 **[49474017]	**BQ13470 **[49474819]
**Length**	**119**	**199**	**280**	**179**

*Bra. jap*.	-	-	-	-
*Rho. pal*.	-	-	-	-
*Agr. tum*.	6e-15 (1.04)	3e-06 (1.07)	2e-07 (1.09)	4e-11 (1.01)
*Sino. meli*.	1e-15 (1.03)	1e-05 (1.03)	1e-11 (1.06)	1e-10 (1.00)
*Bru. mel*.	2e-23 (1.06)	8e-11 (1.01)	1e-17 (1.07)	4e-26 (0.99)
*Bru. suis*	2e-23 (1.06)	1e-11 (1.12)	1e-17 (1.21)	4e-26 (0.99)
*Meso. loti*	3e-12 (1.39)	1e-05 (1.25)	3e-13 (0.96)	3e-24 (1.00)
*Meso. sp*.	2e-12 (1.08)	2e-09 (1.02)	6e-17 (0.95)	8e-09 (0.99)
*B. henselae*	2e-55 (1.00)	1e-64 (0.99)	2e-91 (1.01)	2e-67 (0.99)
*B. Quintana*	1e-64 (1.00)	e-102 (1.00)	e-131 (1.00)	2e-99 (1.00)
Non – Rizobiale	*Bacillus licheniformis *0.77 (1.78)	*Treponema denticola *2.9 (2.32)	*Therm. tengcongensis *0.001 (2.79)	*Mag. mag*. 0.60 (1.04)

See table 1 legend for abbreviations and additional information pertaining to BLAST results. Additional abbreviations: *Therm., Thermoanaerobacter*.

**Table 7:** Signature Proteins specific to the *Bartonellaceae *family.^a^

**Protein**	**BQ12190 **[49474706]	**BQ11460 **[49474647]	**BQ11450 **[49474646]	**BQ11430 **[49474645]	**BQ11380 **[49474640]	**BQ11160 **[49474626]	**BQ11120 **[49474623]	**BQ11100 **[49474621]	**BQ11030 **[49474614]
**Length**	**94**	**103**	**129**	**65**	**76**	**104**	**264**	**231**	**148**

*B. henselae*	2e-27 (1.00)	2e-48 (1.02)	2e-52 (1.00)	3e-22 (1.00)	1e-22 (0.83)	4e-41 (1.05)	e-103 (1.00)	2e-94 (1.00)	3e-64 (0.99)
*B. quintana*	1e-40 (1.00)	3e-52 (1.00)	3e-61 (1.00)	2e-31 (1.00)	4e-38 (1.00)	6e-54 (1.00)	e-145 (1.00)	e-131 (1.00)	4e-83 (1.00)
Other Rizobiales	-	-	-	-	-	-	-	-	-
Non-Rizobiale	Prov. rettgeri 1.0 (2.39)	Symbio. therm. 0.024 (2.16)	Bacillus subtilis 3.9 (3.59)	Lacto. gasseri 2.3 (13.8)	Staph. aureus 0.12 (5.84)	Oceano. iheyensis 1.7 (6.16)	Dehalo. etheno. 0.57 (3.08)	Bacillus cereus 1.7 (1.82)	Citro. freundii 1.8 (5.72)

^a^Abbreviations and further details regarding BLAST results can be found in table 1. Additional abbreviations: *Citro., Citrobacter; Dehalo. etheno., Dehalococcoides ethenogenes; Lacto., Lactobacillus; Oceano., Oceanobacillus; Prov., Providencia; Staph., Staphylococcus; Symbio. therm., Symbiobacterium thermophilum*.

**Table 8:** Other Alpha-proteobacterial Specific Proteins^a^

**Protein**	**CC0189 **[16124444]	**CC0569 **[16124823]	**CC0331 **[16124586]
**Length**	**88**	**288**	**186**

*C. cres*.	5e-38 (1.00)	e-126 (1.00)	e-102 (1.00)
Other Alphas	*Mag. mag*.; 8e-08 (0.93) *Rhod. rubr*.; 8e-08 (0.76) *Nov. aro*.; 1e-10 (0.75) *Sil. pom*.; 2e-09 (0.67) *Sil. sp*.; 2-09 (0.68) *Rh. spha*.; 2e-10 (0.67)	*Nov. aro*.; 1e-40 (1.12) *Meso. loti*; 3e-50 (1.00)	*Bra. jap.; *2e-19 (0.71) Agr. tum.; *2e-23 *(0.87) *Bru. mel*.; 1e-17 (0.76) *Bru. suis*; 2e-23 (0.92) Meso. loti; 6e-20 (0.89)
Non-Alpha	*Nitro. euro*.; 0.089 (0.84)	*Desulf. haf*.; 7e-05 (0.88)	*Micro. deg*.; 0.007 (0.96)
			
**Protein**	**CC0349 **[16124604]	**CC2323 **[16126562]	**CC2637 **[16126872]

**Length**	**265**	**377**	**374**

*C. cres*.	e-138 (1.00)	0.0 (1.00)	0.0
Other Alphas	*Mag. mag*.; 2e-29 (0.98) *Rhod. rubr*.; 8e-32 (0.67) *Nov. aro*.; 3e-44 (0.92) *Sil. sp*.; 5e-27 (0.68)	*Mag. mag*.; 2e-70 (0.98) *Rhod. rubr*.; 2e-68 (1.00) *Bra. jap*.; 3e-40 (1.04) *Rho. pal*.; 4e-36 (1.03) *Agr. tum*.; 7e-23 (1.02) *Sino. meli*.; 3e-27 (1.00) *Meso. sp*.; 7e-23 (1.01)	*Mag. mag*.; 3e-19 (0.99) *Rhod. rubr*.; 1e-23 (1.09) *Bra. jap*.; 7e-22 (0.99) *Rho. pal*.; 8e-19 (0.97) *Agr. tum*.; 2e-04 (0.91) *Sino. meli*.; 8e-13 (1.01) *Meso. loti*; 1e-07 (0.92) *Meso. sp*.; 1e-06 (1.02)
Non-Alpha	*Staph. epi*.; 0.006 (1.34)	*Trep. pallidum*; 0.085 (1.14)	*Pse. fluor*.; 0.32 (1.74)

^a ^Abbreviations and other details regarding BLAST results can be found in Table 1. Additional abbreviations: *Desulf. haf., Desulfitobacterium hafniense; Micro. deg., Microbulbifer degradans; Nitro. euro., Nitrosomonas europaea; Pse. fluor., Pseudomonas fluorescens; Staph. epi., Staphylococcus epidermidis; Trep., Treponema*.

**Table 9:** Alpha-proteobacterial specific proteins with lateral gene transfers. ^a^

**Protein**	**CC2585 **[16126823]	**CC0226 **[16124481]
**Length**	**209**	**132**

Alpha-Proteobacteria	*Mag. mag*.; 2e-16 (0.79) *Rhod. rubr*.; 2e-16 (1.03) *C. cres*.; 2e-96 (1.00) *Rho. pal*.; 7e-21 (1.05) *Agr. tum*.; 7e-19 (1.02) *Sini. mel*.; 2e-19 (1.14) *Bru. mel*.; 5e-19 (1.04) *Bru. suis*.; 5e-19 (1.04) *Meso. loti*.; 6e-35 (1.32)	*Rhod. rubr*.; 8e-14 (0.59) *C. cres*.; 6e-72 (1.00) *Agr. tum*.; 2e-17 (0.64) *Sini. mel*.; 2e-15 (0.64) *Meso. loti*.; 1e-18 (0.75) *Meso. sp*.; 5e-21 (0.64)
Other *Bacteria*	Gamma-proteobacteria: *Az. vine*.; 2e-07 (1.05) *Pse. fluor*.; 3e-07 (1.04) *Pse. aer*.; 1e-06 (1.05) *Pse. syr*.; 1e-06 (1.04)	Gamma-proteobacteria: *Pse. aer*.; 6e-21 (0.64) *Sal. ent*.; 1e-20 (0.64)
Non-Alpha	*Des. vulgaris*; 0.073 (1.40)	*Pro. marinus*; 0.26 (2.54)
		
**Protein**	**CC2790 **[16127022]	**RP382 **[15604247]

**Length**	**567**	**510**

Alpha-Proteobacteria	*Mag. mag*.; 9e-49 (0.41) *Nov. aro*.; 2e-74 (0.78) *C. cres*.; 0.0 (1.00) *Sil. pom*.; 2e-85 (0.72) *Sil. sp*.; 2e-79 (0.76) *Rh. spha*.; 1e-79 (0.74) *Rho. pal*.; 3e-77 (0.68) *Agr. tum*. 6e-82 (0.72) *Bru. mel*.; 1e-86 (0.76) *Bru. suis*;; 6e-83 (0.68) *Meso. sp*.; 2e-28 (1.40)	*R. prowazekii*; 0.0 (1.00) *R. conorii*; e-155 (0.99) *R. akari*; e-141 (1.01) *R. rickettsii*; e-155 (0.99) *R. sibirica*; e-152 (0.99) *Ehr. canis*; 9e-27 (0.65) *Ehr. rum*.; 2e-32 (0.85) *Wolbachia*; 2e-32 (0.82) *Ana. mar*.; 6e-20 (0.85)
Other *Bacteria*	Beta-proteobacteria: *Burk. cepacia*; 4e-18 (0.81)	*Aquificales*: *Aqu. aeolicus*; 7e-11 (0.76)
Non-Alpha	*Strep. coelicolor*; 2.7 (0.65)	*Bac. frag*.; 0.097 (1.00)

^a ^Abbreviations and other details regarding BLAST results can be found in Table 1. Additional abbreviations: *Az. vine., Azotobacter vinelandii; Aqu., Aquifex; Bac. frag., Bacteroides fragilis; Burk. Burkholderia; Des., Desulfovibrio; Lep. int., Leptospira interrogans; Pro., Prochlorococcus; Pse. aer., Pseudomonas aeruginosa; Pse. fluor., Pse. fluorescens; Pse. syr., Pse. syringae; Sal. ent., Salmonella enterica*.

The first grouping of α-proteobacterial markers consists of 6 proteins that are specific to nearly all sequenced α-proteobacterial species and are not found in any other *Bacteria *(Table 1). These proteins clearly distinguish the α-proteobacteria as a distinct group from all other *Bacteria*. Even though some genes have been lost from certain species, these proteins remain largely distinctive of the α-subdivision. Interestingly, no homologues were detected in *Zymomonas mobilis *for three of these signature proteins (CC3319, CC1887, CC1725). *Z. mobilis *is also lacking a number of other signature proteins described in this study and this may be attributed to the genetic loss of a variety genes resulting in its small genome size (2.06 Mb) [33]. A number of genes for the tricarboxcylic acid cycle as well as other functions have previously been documented as missing in this genome [33]. One of these signature proteins (CC1725) is also not found in *Novosphingobium aromaticivorans *indicating it was lost from members of the *Sphingomonadales *family. A homologue of the protein CC3319 was detected in the currently unclassified *Magnetococcus sp. MC-1 *genome suggesting that this species may be distantly related to the α-proteobacteria [42]. A number of α-proteobacteria-specific indels (i.e., inserts or deletions) are also present in *Magnetococcus *[17], supporting the above inference. Finally, the protein CC1887 is also found in the α-proteobacteria as well as a variety of *Eukaryotes *supporting the derivation of mitochondrion from an α-proteobacterial lineage [9-13].

Another group of 10 signature proteins showing a high affinity for sequenced alphas are those distinguishing all other α-proteobacteria from the order *Rickettsiales *(Table 2). In this case, the *Rickettsiales *show no detectable homologues of otherwise α-specific proteins. These results suggests that the genes for these proteins have either been lost from the *Rickettsiales *or it forms one of the earliest branching lineage within α-proteobacteria [2,43]. These proteins are present in almost all other sequenced α-proteobacteria with few exceptions. The proteins CC0520 and CC0366 have homologues in *Magnetococcus sp. MC-1 *again lending support to the inference that this unclassified species is distantly related to the alpha-group. The protein CC1977 is also found in *Eukaryotes *and the E values for a few representative eukaryotic species are given in the Table 2 legend. One protein (CC3010), showing a very high affinity for this grouping as noted by low E values, is also found in a single gamma proteobacterium (*Pseudomonas sp*.). This finding is most likely due to a non-specific event such as a lateral gene transfer (LGT) of which additional examples will be presented later.

The next grouping of signature proteins are those which are found in almost all sequenced α-proteobacteria excluding the intracellular pathogens belonging to the *Bartonellaceae *family and the order *Rickettsiales *(Table 3A). This grouping outlines a case in which proteins have probably been lost independently from two unrelated groups within the α-proteobacteria most likely due to their intracellular lifestyles [2,3,44]. Five proteins of this type were identified with minimal loses seen in other α-proteobacteria. CC2345 provides a good example of this type of protein since it is highly conserved in all available α-proteobacterial genomes as indicated by low E values. The other four proteins also show a high affinity for this category with losses occurring only in *Z. mobilis *and *Rhodospirillum rubrum*.

A variation on the above theme is a collection of 4 α-specific proteins that are absent in the orders *Rickettsiales *and *Rhodobacterales *(Table 3B). However, a key feature distinguishing these proteins from those presented in Table 3A is the free-living lifestyle of the *Rhodobacterales *as opposed to the intracellular *Bartonellas*. Since *Rickettsiales *and the *Rhodobacterales *are not known to share any unique characteristic, it is possible that the loss of these proteins from these two orders has occurred due to unrelated reasons. Also, some additional losses are seen in this grouping. For example the protein CC1652 is absent in the *Sphingomonadales *while the protein CC1035 is absent in the *Rhodospirillales*. Note that the protein CC2247 exhibits high E values for BLAST hits representing *Brucellaceae *and *Bartonellaceae *but this high E value is acceptable due to the very short length of this protein (46 amino acids) and the fact that besides α-proteobacteria no other BLAST hits were observed (Table 3B).

The Blast searches on proteins found in the *R. prowazekii *genome have led to identification of a number of signature proteins which are specific to species belonging to the order *Rickettsiales*. This order is made up of two families: the *Anaplasmataceae *(*Anaplasma, Ehrlichia and Wolbachia*) and *Rickettsiaceae *(*Rickettsias*) [2,43]. The first group of such proteins (RP104, RP105, and RP106) are present in all species belonging to the order *Rickettsiales*, but are not found in any other α-proteobacteria (Table 4). It should be noted that the proteins RP104 and RP106 do not show homology over the entire length of the homologous proteins in members of the *Anaplasmataceae *family. Thus, additional domains that are specific for the *Rickettsiaceae *family may be present in these proteins. These signature proteins are highly conserved within this order, as indicated by their very low E values (Table 4) and represent interesting examples of genes that were likely introduced in a common ancestor of the *Rickettsiales*. Note that the first non-*Rickettsiale *BLAST hit for the protein RP106 appears at 2e-07 (*Xyella fastidiosa*). RP106 is still included as a *Rickettsiales*-specific protein because the *Xyella *protein is only 348 amino acids in length and thus it is likely a different protein.

Another group of 4 proteins are specific to the *Rickettsia *species and are not found in other members of the *Rickettsiales *(Table 4). These proteins (RP766, RP192, RP030 and RP187) are highly conserved and represent cases in which genes were introduced into a common ancestor of the *Rickettsiaceae*. Homologues of the protein RP187 are much longer in other *Rickettsia *strains (194 vs 497 aa) but the region representing the query sequence is highly conserved. It is possible that other *Rickettsia *species have acquired an additional protein domain during the course of evolution.

In addition to the *Rickettsiales*, the *Rhizobiales *form a major order within the α-proteobacteria [1,17,42]. To identify proteins which are distinctive of the *Rhizobiales*, BLAST searches were carried out on all ORFs in the genome of *B. quintana*. Six proteins have been identified that are conserved amongst all sequenced *Rhizobiales *with minimal evidence of gene loss occurring (Table 5). The protein BQ07670 is absent in *Rhodopseudomonas palustris *while the protein BQ11900 is absent in this strain as well as in *Sinorhizobium meliloti*. The presence of these proteins solely in the *Rhizobiales *indicates they were likely introduced in a common ancestor of this order.

Other signature proteins that are useful in defining the *Rhizobiales *are those which are present in all sequences members of this order, except the *Bradyrhizobiaceae *family (Table 6). Four proteins of this type have been identified with no losses occurring in any species. These proteins indicate that the *Bradyrhizobiaceae *family is more distantly related to other members of the *Rhizobiales*. The deeper branching and distinctness of *Bradyrhizobiaceae *and *Methylobacteriaceae *from other *Rhizobiales *is also strongly supported by phylogenetic analyses based on different gene sequences and conserved indels in many proteins [1,17,45].

A number of proteins have also been identified which are unique to the *Bartonella *species. Nine examples of such proteins are shown in Table 7. These proteins are highly conserved amongst both sequenced *Bartonella *species with no gene losses occurring. The presence of these proteins solely in this family of α-proteobacteria indicates that they should provide useful markers for the *Bartonellaceae *family.

Six other α-specific signature proteins were identified that do not show any distinct pattern but are sporadically present in α-proteobacterial species (Table 8). These proteins are more randomly distributed among a limited number of sequenced α-proteobacteria and it is likely that gene losses for these proteins have occurred independently in various species or groups. Nevertheless, these proteins are still unique to the α-proteobacteria. The protein CC0189 is represented in the *Rhodospirillales, Novosphingomonadales, Caulobacterales *and *Rhodobacterales *but is not found in any *Rhizobiales*. One protein (CC0331) is represented in various families within the *Rhizobiales *while two others (CC2323 and CC2637) show a similar trend and are also present in *Rhodospirillales*.

A final grouping of 4 signature proteins consists of those where limited lateral gene transfers (LGTs) have apparently occurred (Table 9). Three of these proteins (CC2585, CC0226 and CC2790) were isolated from the *Caulobacter *genome and represent cases in which genes were also present in a limited numbers of gamma or beta-proteobacteria. Specifically, a homologue of the protein CC2585 was detected in a number of gamma-proteobacteria belonging to the *Pseudomonadaceae *family while CC0226 was only detected in *Pse. aeruginosa *and the enteric bacterium *Salmonella enterica*. The protein CC2790 shows some similarity to a Superfamily I DNA and RNA helicase found in *Burkholderia cepacia *(beta-proteobacteria). However, this BLAST hit only shows conservation over 142 amino acids of the 567 amino acids *C. crescentus *protein. Furthermore, all alpha BLAST hits are annotated as hypothetical proteins indicating this non-alpha BLAST hit probably represents a different protein with a shared protein domain that was transferred. Interestingly, one of the proteins, RP382, which is otherwise highly specific for the order *Rickettsiales*, is also found in *Aquifex aeolicus*. In each of these cases, the direction of gene transfer remains unclear.

## Discussion

**Figure 1:**
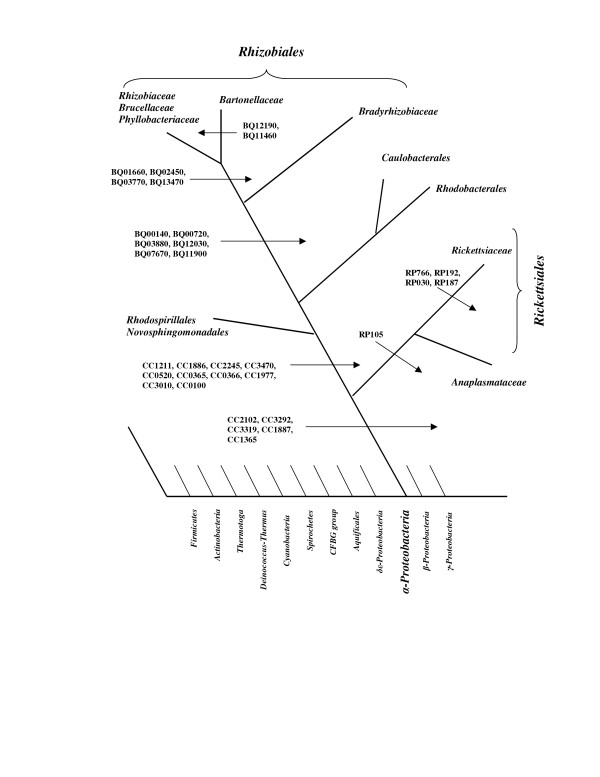
Summary diagram showing the distribution pattern of various α-proteobacteria signature proteins. The arrows indicate the evolutionary stages where these signature proteins were likely introduced. Some proteins, which are sporadically present in α-proteobacteria are not shown here. The branching position of α-proteobacteria relative to other bacterial groups was deduced as described in earlier work [13,17,40].

The α-proteobacteria forms an extremely diverse group showing vast differences in such characteristics as morphology, metabolism, and physiology [1]. In the current view, this group is distinguished from all other *Bacteria *based on 16S rRNA phylogenetic trees [1,8,19,46]. Few molecular or physiological characteristics were known which clearly distinguish this group from all other *Bacteria *[1,7]. However, our recent work has identified a large number of conserved inserts and deletions in protein sequences which are distinctive characteristics of α-proteobacteria and its subgroups and not found in any other groups of *Bacteria *[17] (see also ). These signatures provide useful tools for identifying α-proteobacteria within *Bacteria *as well as for understanding the interrelationships and branching order within this group. Here, we describe 61 signature proteins that are largely specific for the α-proteobacteria. Almost all of these proteins are of hypothetical functions, and in view of their α-proteobacterial specificity, it is likely that they are involved in functions that are limited to only this group of bacteria. Because such genes are likely involved in specialized functions, the loss of some of these genes from certain α-proteobacterial species is not surprising. Based on signature proteins described here, along with various α-proteobacteria-specific conserved inserts and deletions [17], a clearer picture of α-proteobacteria phylogeny and taxonomic classification can be derived. Figure 1 presents a model for α-proteobacterial evolution which indicates the evolutionary stages where these proteins are suggested to have evolved or been introduced. The model based on these signature proteins is identical to that deduced independently based upon a large number of conserved indels in different proteins [17], indicating its reliability.

Several signature proteins are specific to nearly all α-proteobacteria. These proteins provide additional support to various alpha-distinguishing indels, which are found only in the α-proteobacteria and not in any other groups of bacteria. Examples of such indels include the following: an 8 amino acid insert in the α subunit of ATP synthase complex, 3 amino acid insert in prolipoprotein-phosphatidylglycerol transferase, and a 1 amino acid deletion in the FtsK protein [17]. The simplest and most parsimonious explanation for the presence of these α-specific signatures (both proteins and indels) is that they were introduced once in a common ancestor of all α-proteobacteria and their presence in various α-proteobacterial species is due to vertical transmission [47,48]. It is difficult to explain the presence of these genes in various α-proteobacteria by other non-specific means such as lateral gene transfers [49]. The finding of these unique genes and conserved indels in various α-subdivision members strongly indicates that all such bacteria carry out certain physiological functions that are unique to the members of this group. Therefore, studies aimed at determining the functional roles of these proteins and indels are of much interest.

The largest group of signature proteins discovered are those found in all α-proteobacteria excluding the order *Rickettsiales*. These proteins indicate that the *Rickettsiales *constitute a distinct clade within the α-subdivision, which is in accordance with phylogenetic analyses based on different gene sequences [2,17,43,50]. Phylogenetic studies based on 16S rRNA and many other genes [2,43,45,50], as well as our studies based on conserved indels in several proteins that are present in various α-proteobacteria but absent in *Rickettsiales *as well as other groups of bacteria [17], provide evidence that the *Rickettsiales *comprise the deepest branching group within α-proteobacteria. In view of this, the most logical explanation for these signatures is that they were introduced in a common ancestor of other α-proteobacteria after the divergence of the *Rickettsiales *(Figure 1).

An interesting group of α-specific signature proteins are those which are absent in the intracellular pathogens belonging to the order *Rickettsiales *and the family *Bartonellaceae*. The latter group of species form a family within the *Rhizobiales *order [1,17]. Because these two groups are phylogenetically unrelated, it is likely that the genes for these proteins were selectively lost in these two groups independently due to their intracellular lifestyles. It is logical to assume that the cellular functions of these proteins are either not required in the intracellular environment, or they are provided for by the host cells leading to the loss of these genes from these organisms. These proteins could have been introduced in either a common ancestor of all α-proteobacteria and subsequently lost in the *Rickettsiales *and *Bartonellaceae*, or introduced after the divergence of the *Rickettsiales *and lost in the *Bartonellaceae*. It is interesting that the *Brucellas *(also intracellular pathogens) have retained all of these proteins indicating that this group differ in its physiological requirements from other α-proteobacterial intracellular pathogens [1,3,51]. Several α-specific signature proteins that are absent in both the *Rickettsiales *as well as *Rhodobacterales *were also identified. Since there is no evidence to suggest any sort of relationship between these two groups [1,17], the simplest explanation is that these genes were introduced after the divergence of the *Rickettsiales *and lost preferentially by the *Rhodobacterales*.

Other signature proteins were isolated pointing to a variety of relationships. For instance, the protein CC0189 which is only present in *Caulobacterales, Rhodobacteriales, Rhodospirillales *and *Novosphingomonadales *indicates a close relationship between these deep branching orders within α-proteobacteria. This relationship is also seen from the protein CC0349 but to a lesser extent since losses have occurred in some species. These findings are supported by indels in a variety of proteins that indicate these orders show a closer relationship and have branched prior to the *Rhizobiales *[17]. Other signature proteins are found in a selection of these above orders and are also found in some but not all families within the *Rhizobiales *(CC0331, CC2323 and CC2637). A close relationship between *Caulobacter *and *Rhodobacterales *is generally indicated by phylogenetic trees and is also supported by a conserved 11 amino acid insert in the protein aspargine-glutamine amido transferase [1,17]. Thus, it is somewhat surprising that in our analysis of the *Caulobacter *genome, we did not identify any signature protein that was uniquely shared by these two α-proteobacterial orders. However, a 12 amino acid insert in the protein DNA ligase indicates that *Rhodobacterales *may be more closely related to *Rhizobiales *in comparison to *Caulobacterales *[17]. In view of these results, and the fact that *C. crescentus *represents the only fully sequenced bacterium within its order [24], additional sequence information is required to further clarify the evolutionary relationships amongst *Rhizobiales, Rhodobacterales *and *Caulobacterales*.

Several signature proteins were found to be specific for either the order *Rickettsiales *or the family *Rickettsiaceae*. These proteins provide molecular markers for these groups and they likely originated in common ancestors of these groups. The distinctness of these groups is also supported by a number of conserved indels in different proteins which are uniquely present in the species from these groups, but not found in any other bacteria [17]. It should be noted that McLeod et al. [28] based upon their comparative analysis of the *Rickettsias *genomes have identified a number of hypothetical proteins that are only found in particular *Rickettsias*. These proteins were grouped into the following classes: *R. typhi *ORFs not found in *R. conorii *or *R. prowazekii*; *R. typhi *ORFs found in *R. conorii *but not in *R. prowazekii*; and *R. typhi *ORFs found in *R. prowazekii *but not in *R. conorii*. However, no proteins that were specific for all *Rickettsias *or *Rickettsiales *were described in the McLeod et al. study [28].

A number of signature proteins identified here are useful in defining and characterizing the *Rhizobiales *order. Of the six *Rhizobiales*-specific proteins described here, four (viz. BQ00140, BQ00720, BQ03880 and BQ12030) are completely conserved amongst all sequenced *Rhizobiales *and should provide good molecular markers for this order. Two other proteins (BQ07670 and BQ11900) also show a high affinity for this grouping with a few gene losses in some species. We have previously described a conserved indel in tryptophanyl-tRNA synthetase that is present in all sequenced *Rhizobiales *but is absent in all other bacteria [17]. These signatures were likely introduced in a common ancestor of the *Rhizobiales *order (Figure 1). Four additional proteins that were identified here are completely specific to all sequenced *Rhizobiales*, except for the *Bradyrhizobiaceae *family. Phylogenetic analysis based on a number of gene sequences as well as conserved indels in a number of proteins (viz. Trp-tRNA synthetase, LytB metalloproteinase) provide evidence that that the *Bradyrhizobiaceae *family is distantly related to other *Rhizobiales *(*Rhizobiaceae*, *Brucellaceae*, *Phyllobacteriaceae*), and it has branched prior to the latter groups of species [1,17,45]. Thus, it is likely that these signature proteins evolved in a common ancestor of various other *Rhizobiales *after the divergence of the *Bradyrhizobiaceae *family (Figure 1). A number of signature proteins that are unique for the *Bartonella *species were later introduced in that particular branch of the tree (Figure 1).

Although most of the signature proteins identified here are specific for only the α-proteobacteria, we have also come across a few examples where lateral gene transfer seems to have occurred between α-proteobacteria and a few species from other groups of bacteria. The rarity of such proteins in comparison to those which exhibit strict group-specificity indicates that most newly acquired alpha-specific genes have been predominantly transmitted via vertical descent and LGT and other non-specific mechanisms play relatively minor role in their transmission. It should be mentioned that although our analyses of proteins in *R. prowazekii, C. crescentus *and *B. quintana *genomes have identified a large number of signature proteins, based on these studies signature proteins for certain other groups within α-proteobacteria (e.g. *Rhizobiaceae, Rhodobacterales, Sphignomonadales*, etc.) will not be detected. It should be possible to identify signatures for these groups by carrying out similar analysis using protein sequences from these genomes.

Daubin and Ochmann [52] and Lerat et al. [36,47] have previously examined the gene repertoire of γ-proteobacteria and have indicated the presence of many ORFans genes (i.e. ORFs that have no known homologs) that are limited to either certain bacterial strains or particular subgroups of γ-proteobacteria. The ORFan genes were found to be present in their studies in different monophyletic clades at different phylogenetic depths, which is similar to what we have reported here for the signature proteins in the α-proteobacteria taxon. The other characteristics of ORFans genes noted by these authors were that they are generally short (between 400–500 bp), A+T rich, and evolve faster than other genes which are more broadly distributed [47,52]. Many of the signature proteins identified in the present work are of similar lengths as the ORFans genes. These earlier studies also indicate that ORFans genes generally encode for functional proteins, and once acquired they are vertically transmitted, and based on them it possible to make robust phylogenetic inference as we have been able to do in the present study for α-proteobacteria. Although the source of ORFans genes in different genomes remains to be determined, it has been suggested that many of them are derived from bacteriophages [47,52].

The concept that mitochondria have originated from an α-proteobacterial ancestor is supported by a large body of evidence including phylogenetic analysis and shared presence of many common indels [9-14]. The homologues of two of the α-proteobacterial signature proteins (CC1887 and CC1977) are also present in *Eukaryotes *providing further support for this inference. For the remainder of the proteins no eukaryotic homologues were detected which supports the observation of Boussau et al. [44] that for a large fraction of genes in α-proteobacterial genome no homologs are found in the eukaryotes. Currently, it is thought that within α-proteobacteria the species belonging to the order *Rickettsiales *are the closest relatives of mitochondria [10-12,53-55]. However, of the two proteins which are commonly found in eukaryotes, only one of them (CC1887) is present in the *Rickettsiales*. A specific relationship of mitochondria to the *Rickettsiales *is also supported by only some conserved indels, but not all [17]. In a recent study, where the relationship of alpha proteobacteria to mitochondria was examined based on a large number of individual and concatenated protein sequences [56], the closest relationship of mitochondria was seen for *Rhodospirillum rubrum *rather than the *Rickettsiales*. In earlier work, we have described two conserved signatures (a 37 aa insert in valyl-tRNA synthetase and 1 aa indel in LonA protein), which were commonly shared by all eukaryotic homologs and certain other groups of bacteria but which were not found in any α-proteobacteria [13]. An update of these signatures indicates that they still constitute exceptions to the α-proteobacterial derivation of the mitochondrial/eukaryotic homologs (R.S. Gupta, unpublished results). These observations in conjunction with the recent conflicting observations regarding the possible origins of NADH dehydrogenase subunits from *Trichomonas vaginalis *[57,58] indicate that additional work is necessary to clarify the sources of different mitochondrial and nuclear cytosolic genes of eukaryotic proteins.

## Conclusion

Whole-genome analyses of *B. quintana, Ri prowazekii *and *C. crescentus *proteins have led to the discovery of 61 signature proteins which are distinctive characteristics of the α-proteobacteria and its subgroups. These signature proteins provide additional support to our recent work based on a large number of conserved inserts and deletions in protein sequences that are either specific for the α-proteobacteria or provide information regarding the interrelationships and branching order within this group [17]. Sequence information from additional α-proteobacterial species will be useful in testing the predicted presence or absence of various identified molecular signatures (indels and proteins) in different groups, thus validating the suggested relationships. Studies aimed at understanding the cellular functions of these α-specific signature proteins should be of much interest since they will likely provide novel insights into unique physiological characteristics shared by this important group of bacteria and its various subgroups. Studies on proteins which are specific to the intracellular pathogens, such as *Rickettsiales *and *Bartonella*, could also provide new drug targets for their associated diseases.

## Methods

### Identification of α-Proteobacteria Specific Proteins

To identify signature proteins which are specific to the α-proteobacteria or its various subgroups, all proteins in the genomes of *C. crescentus, R. prowazekii*, and *B. quintana *were analyzed. BLAST searches were carried out [41] on each individual protein in these genomes to identify all other bacteria containing proteins with similar sequences. These results were visually inspected for homologues showing specificity to α-proteobacteria with no other similar homologues present in any other *Bacteria*. Expect values (E values) were analyzed for putative α-specific proteins. The E values, which are calculated by the BLAST software, indicate the probability that the observed similarity between the query protein and any other protein detected by the BLAST search arose by chance [41]. In BLAST searches, the E values are lowest (closer to 0) for BLAST hits with a high degree of homology to the query sequence and they increase as BLAST hits are detected with lower similarity. The results of BLAST searches were inspected for sudden increases in E values from the last α-proteobacteria in the search to the first non-alpha bacteria. This increase in E values was important when the next non-alpha BLAST hit was in a range where the observed similarity could occur by chance (> 10^-05^). However, higher E values were sometimes allowed and could be significant for smaller proteins since they contain fewer characters resulting in higher E values (for statistical reasons) for their true homologs. For all α-specific signature proteins described here, E values were recorded for each blast hit as well as for the first non-alpha bacterium in a given search. Although E values take into account the length of the sequence over which the similarity is observed between the query sequence and a BLAST hit, low E values can sometime result if high degree of homology is observed between two different proteins over a short sequence region. Therefore, we have also inspected BLAST results for homology over the entire protein length and for similarity in protein length. The length ratios of the hit proteins over the query protein are shown in brackets beside the E values and these values are expected to be close to 1.00 if the identified proteins are of similar lengths as the query protein. It should be mentioned that BLAST searches can sometime indicate misleading similarity, particularly when no close relatives of the query species are in the database [59]. However, in the present study where most of the BLAST hits correspond to α-proteobacteria, such a possibility is highly unlikely. All proteins indicated in the Tables 1, 2, 3, 4, 5, 6, 7, 8, 9 are specific for the α-proteobacteria based on these criteria unless otherwise mentioned.

## Authors' contributions

PK carried out BLAST searches on different proteins and was responsible for the initial evaluation of the results. RSG conceived and directed this study and was responsible for the final evaluation of the results. PK prepared a rough draft of the manuscript under RSG's directions, which was revised and modified by RSG. All authors read and approved the final manuscript.
